# Exploring models for the roles of health systems’ responsiveness and social determinants in explaining universal health coverage and health outcomes

**DOI:** 10.3402/gha.v9.29329

**Published:** 2016-03-01

**Authors:** Nicole Britt Valentine, Gouke J. Bonsel

**Affiliations:** 1 World Health Organization, Geneva, Switzerland; 2 Department of Public Health, Erasmus University Rotterdam, Rotterdam, The Netherlands; 3 Division Mother & Child, University Medical Center Utrecht, Utrecht, The Netherlands

**Keywords:** health information, health determinants, health systems, gender

## Abstract

**Background:**

Intersectoral perspectives of health are present in the rhetoric of the sustainable development goals. Yet its descriptions of systematic approaches for an intersectoral monitoring vision, joining determinants of health, and barriers or facilitators to accessing healthcare services are lacking.

**Objective:**

To explore models of associations between health outcomes and health service coverage, and health determinants and health systems responsiveness, and thereby to contribute to monitoring, analysis, and assessment approaches informed by an intersectoral vision of health.

**Design:**

The study is designed as a series of ecological, cross-country regression analyses, covering between 23 and 57 countries with dependent health variables concentrated on the years 2002–2003. Countries cover a range of development contexts. Health outcome and health service coverage dependent variables were derived from World Health Organization (WHO) information sources. Predictor variables representing determinants are derived from the WHO and World Bank databases; variables used for health systems’ responsiveness are derived from the WHO World Health Survey. Responsiveness is a measure of acceptability of health services to the population, complementing financial health protection.

**Results:**

Health determinants’ indicators – access to improved drinking sources, accountability, and average years of schooling – were statistically significant in particular health outcome regressions. Statistically significant coefficients were more common for mortality rate regressions than for coverage rate regressions. Responsiveness was systematically associated with poorer health and health service coverage. With respect to levels of inequality in health, the indicator of responsiveness problems experienced by the unhealthy poor groups in the population was statistically significant for regressions on measles vaccination inequalities between rich and poor. For the broader determinants, the Gini mattered most for inequalities in child mortality; education mattered more for inequalities in births attended by skilled personnel.

**Conclusions:**

This paper adds to the literature on comparative health systems research. National and international health monitoring frameworks need to incorporate indicators on trends in and impacts of other policy sectors on health. This will empower the health sector to carry out public health practices that promote health and health equity.

## Introduction

In the first decade of the twenty-first century, the World Health Organization (WHO) played a leading role in harmonizing health systems’ performance assessment approaches through the development of relevant conceptual frameworks (1–4). These frameworks refer to five health system goals that are achieved through the intermediate goal of coverage of the population with needed health services. According to these frameworks, different combinations of health systems’ functions such as stewardship, financing, or service delivery can be evaluated based on how well they improve the intermediate and final goals. The final goals are improvements in: population health levels, population health equity, levels of health systems’ responsiveness to the legitimate expectations of the population, responsiveness equity, and fairness in financial contributions. When considering these frameworks and the associated monitoring approaches they have generated in the context of the sustainable development goals (SDGs) (5, 6), one can make several observations concerning potential areas for improvement. We focus on two areas for improvement for the purposes of this paper.

A first area for improvement in these frameworks is to address the neglect of the critical role of determinants beyond the health sector on population health. From the leading nineteenth century German doctor, Rudolf Virchow, to the present-day discussions on sustainable development, there is general recognition that average levels of population health and health inequities arise from factors beyond health care and the health systems’ direct control. This implies augmentation of the original WHO frameworks mentioned above to include causal pathways beyond service coverage. As Hippocrates observed, social and environmental factors affect health directly. Yet social and environmental factors may give rise to additional problems with access to health services, thus modifying or even augmenting their direct effects on population health.

In order to be comprehensive and efficient, health performance frameworks and associated monitoring should track trends in these broader determinants. This will allow the health sector to detect, understand, influence, anticipate, and possibly even alter the health impacts of decisions in other sectors. The WHO Commission on Social Determinants of Health argued in 2008 that impacts of health determinants, in particular social determinants related to the distribution of power, money, and resources, were even more important for addressing health equity (7).

A second area for improvement relates to the development of measures of non-financial barriers to access to health services. We use the term ‘non-financial’ to distinguish a set of barriers that complement the financing of direct medical expenses. The so-called non-financial barriers may have components related to indirect costs (e.g. food, fear of loss of income), but also include other barriers related to acceptability and access (e.g. treatment with dignity and non-discrimination). Non-financial barriers to health service access are related to health determinants. For example, the lack of transport in rural areas may result in longer travel distances to health facilities and differential health service access for disadvantaged groups. At the same time, the lack of transport can affect access to work with direct impacts on health through reducing family time or the length of periods of breastfeeding.

Although non-financial outcomes of health systems were reflected in WHO's original frameworks by the concept of health systems’ responsiveness, advances in routine application in measurement and monitoring have been slow. Responsiveness is the degree to which legitimate expectations of the population with respect to non-clinical aspects of health care or public health services were actually met (1). It is measured through large representative general population household surveys, or targeted surveys among recent care users. The responsiveness domains are, in alphabetical order: autonomy, choice, communication, confidentiality, dignity, prompt attention, (quality of) basic amenities, and (access to family and community) social support. The work of Donabedian, Tanahahsi, and others suggest that responsiveness has a direct positive relationship with service coverage and the final target, health (8–11).

We therefore plead for a broader measurement and monitoring framework, incorporating responsiveness and determinants, to be applied to evaluating health systems performance. To investigate the case for this empirically, this paper describes the development of analytical models that use data on health systems responsiveness and indicators of social and environmental determinants of health for their association with key outcomes from the original WHO frameworks. These key outcomes relate to average levels of population health and health equity and the intermediate goals of health service coverage and service coverage equity. These outcomes of interest are important in light of the SDGs, as several measures of average levels of health and universal health coverage (UHC) have been accepted as part of the SDGs monitoring framework (12). In our paper, responsiveness and determinants are evaluated in terms of their instrumental contribution to health and health service coverage.

This paper investigates the association between population health outcomes, UHC, and responsiveness, and the role of determinants. We explore regression models, variables, and country-level indicators for determinants and responsiveness using cross-sectional data for between 23 and 57 countries. We observe whether a small basket of theory-supported determinants indicators explain expected linkages at the ecological level to health and coverage outcomes.

## Methods

The approach was: 1) to define a hypothesis-driven set of variables representing health service coverage, health, health systems responsiveness, health systems financial protection, and broader societal factors referred to as health determinants, suitable to test relationships; 2) to select and link accessible data sets for testing; and 3) to conduct multiple regression analyses to assess the hypothesized associations. The country set was confined to those listed in the 57 face-to-face complete surveys of the World Health Survey (WHS) (2002–2003; see Appendix 1), for which comparable health systems’ responsiveness information is available.

### Model

**Fig. 1:**
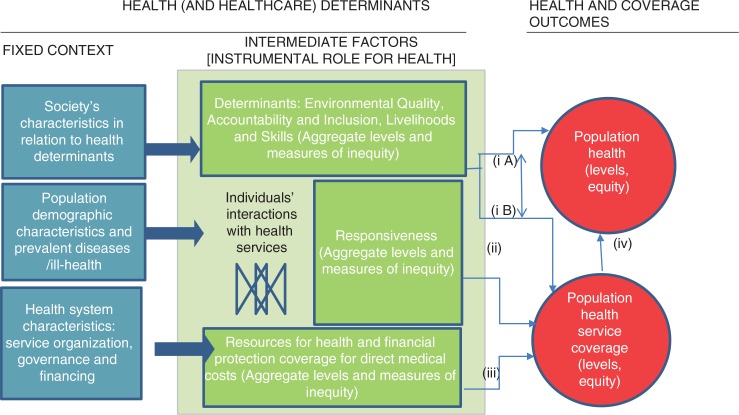
Analytical model for tracing key pathways of influence determinants and responsiveness on population health and population health service coverage.

The analytical model that underpins the variables and regression analyses of population health and service coverage in this paper is represented in Fig. 1. This describes the main pathways related to how determinants and responsiveness, as instrumental variables, affect population health and service coverage. It is derived from standard literature of conceptual models or frameworks related to how the broader society interacts with health systems to ‘produce’ health (7, 13–15). In view of the broader analytical focus of this paper at this stage, these broader conceptual frameworks were considered to provide more relevant starting points than frameworks for monitoring of health services (e.g. monitoring of UHC). The societal-level models tend to show the role of determinants quite strongly. The unique feature we added is the separate, instrumental and therefore testable role given to responsiveness, assuming that people-centredness matters for health and coverage outcomes, thus representing a pathway in itself.

From left to right, Fig. 1 describes the pathways of influence on population health and universal population health service coverage. Starting first with the blue block, the analytic framework assumes that ‘fixed’ characteristics of population, society, and the health systems’ functions determine the context for intermediate factors that are more directly associated with health and coverage. These ‘fixed’ characteristics (left) are considered unchangeable for a given period of time and result in multiple influences on intermediate factors. The intermediate factors shown in the centre column operate at the individual-level and include exposures or access to health services, for which empirical studies have shown more direct causative associations with population health (average levels and health equity) and health service coverage (average levels and coverage equity) (on the right) (7). The intended analyses focus on the pathways (i) determinants to health and coverage (not distinguishing between iA and iB); (ii) responsiveness to coverage; and (iii) financial resources and financial health protection to coverage. Pathway (iv) is assumed as implicit. Given the importance of financial protection for health service coverage, it was necessary to model it, although the focus of the paper is on the additional roles of determinants and health systems responsiveness. Below we elaborate several generic implications of this analytical model for structuring the analyses that follow.

#### Health outcomes

1. Two outcomes should be considered in the regression models in order to cover two separate but important measures of health systems performance: population health and coverage of the population with essential health services (population health service coverage).
2. The outcome measures tested as the dependent variables should cover a spectrum of disease profiles and health service interventions.
3. Equity measures of these main coverage and health outcomes should also be considered in order to assess specific pathways for inequities in health and coverage.

#### Health determinants

1. Determinants as intermediate factors can be measured at the individual-level and aggregated to the country-level, but they can also be measured by policy variables.
2. Distributional measures of determinants measured at the individual-level should be tested for modelling equity in health and health service coverage.
3. Health systems are key determinants of health and health service coverage. Proxy measures of health systems should be included in regression models and should relate to levels of resources and financial protection, given its importance as a determinant of health service clinical quality and access (5).

The next sections focus on scoping recommended dependent and independent variables outlined in the analytical model and on selecting the final data set for the regression models.

### Scope of variables

#### Health and coverage (dependent variables)

Population health status can be represented by rates of morbidity, mortality, the compound indicator life expectancy, or self-reported health. Health service coverage can be characterized by enrolment, utilization, or effective service coverage rates (population in need receiving treatment divided by the population in need). Coverage rates can be measured comparably if morbidity–intervention combinations are standardized across countries (5). Similar to other studies (16), the following additional criteria were applied to select the final set of dependent variables: 1) completeness of the data for the time period and countries; 2) a spectrum of health conditions or interventions covering reproductive, maternal and child health, communicable diseases (the so-called ‘unfinished’ millennium development goals), and non-communicable diseases and injuries; and 3) variables for which country-level inequality data were available. Inequalities in health outcomes can be measured as gaps or concentration measures describing between-group differences in aggregate health outcome levels, where groups are defined by a ‘wealth’ or ‘income’ quintile (e.g. absolute or relative gap between fifth and first quintiles), sex, geographic areas, and educational attainment (17).

#### Predictor and control variables (independent variables)

*Health determinant* variables (that are under the control of policy sectors other than the health sector) can be conveniently grouped into the following categories environmental quality, accountability and inclusion, and livelihoods and skills (referred to as EQuAL). A variant of these categories was discussed at an expert meeting held by WHO (18). Based on these categories, a range of potentially relevant country-level indicators were drawn from a descriptive review of recent peer-review literature and from key informant reports.

*Environment quality* indicators representing physical exposures are: urban households living in ‘durable’ structures; population exposed to small/fine urban particulates (PM10 or PM2.5) in concentrations exceeding WHO Air Quality Guidelines; households using modern fuels/technologies for all cooking, heating, and lighting activities; health facilities with access to clean and reliable electricity; population using a basic (improved) water source; the population whose access to safe water sources and sanitation is at risk from changing climate (19, 20); exposure to harmful substances in the work environment, and broader physical conditions in the work environment (e.g. night shifts, length of working week). Social elements of housing are: residential stability or affordability of neighbourhoods (20); urban design or green space and safety, and for products, enforceable and regulatory product quality and labelling measures (21).

*Accountability and inclusion* indicators include: violence against women; ratios of female to male schooling (attainment); social capital; self-reported gender inequality or discrimination; discrimination in laws and policies, and related composite indices (e.g. World Bank Good Governance database) (22).

*Livelihoods and skills* indicators include: child stunting (23); caloric intake; household poverty; access to social protection (e.g. cash transfers); value in work; associated psychosocial exposures; employment relations (e.g. informal or formal, own account/salaried – access to paid parental leave, old age pensions); maternal education and birth spacing; child development; access to early child development services, and social inequality (18, 23).

*Responsiveness* measurement is described in the literature (24, 25). The original eight responsiveness domains can be regrouped by the EQuAL framework: basic amenities and communication under Environmental quality; autonomy, confidentiality, dignity, and social support under Accountability and inclusion, and choice and prompt attention under Livelihoods and skills.

*Health systems* pathways related to health system availability and financing are characterized in terms of levels of expenditure and financial protection coverage (other factors less commonly considered are human resource levels) (4, 16). Out-of-pocket expenditure indicators often represent financial protection coverage, with higher levels representing higher copayments or low financial protection coverage (16, 26), which are known to be regressive (27).

*Demographic and biological drivers of need* are primarily age and sex structure of the population. For our analysis, such variables are controlled for as has been done elsewhere (28).

### Data sources and final data sets

#### Country-level indicators and data

In view of the scope of variables and indicators outlined above, we scanned the range of potential data sources from WHO (World Health Statistics; Global Health Observatory) and World Bank (World Development Indicators including the Worldwide Governance Indicators) databases. Country-level data were obtained, in most cases for the years 2002–2003. For responsiveness, we needed to calculate country-level measures from individual-level data sets from the WHS. The WHO WHS data are the only large publicly available cross-country and region source with information on a range of health system responsiveness domains. Implemented between 2002 and 2004, the WHS data, acquired through nationally representative and quality-controlled surveys, have been widely used in the peer-review health literature (29). Its data on responsiveness cover 57 countries and 151,848 respondents (using public and private sector providers). The selection of the remaining indicators was made for these 57 countries classified by the United Nations Development Agency in 2003 (30): 23 low-income countries; 13 lower middle-income countries; 11 upper middle-income countries; and 10 high-income countries.

Table 1 lists the final indicator names, the number of observations obtained, descriptive statistics, and data sources (31–34). All data except for responsiveness were obtained as country-level indicators. The estimation of country-level responsiveness indicators from the World Health Survey individual-level data set (35) is described in detail below.

**Table 1:** Variables used in regression models: descriptive statistics and data sources

			Descriptive statistics				Data source	
		
Analytic model categories	Variable or indicator names		Mean	Std dev	Minimum	Maximum	Reference	Year
Population health levels	All (*n*=57)							
	Maternal mortality per 100,000 live births (2005)		308	368	1	1,500	(31)	2005
	Under 5 child mortality per 1,000 live births (2005)		63	66	4	220	(32)	2005
	TB cause of death per 100,000 (2004)		36	50	0.5	269	(33)	2004
Population health service coverage levels	All (*n*=52)							
	Percentage of births attended by skilled health personnel (2000–2006)		76	28	6	100	(31)	2000–2006
	Percentage of population covered with 1 dose of measles vaccination (2003)		84	15	42	99	(34)	2003
	Percentage of women receiving a Pap smear (2000–2006)		31	29	0.1	82	(31)	2000–2006
Population health and service coverage equity^a^	All (*n*=23)							
	Child mortality: absolute difference by wealth quintile [poor quintile (I)/less wealthy quintile (V)]		−57.6	32.8	−157	−15	(31)	1996–2006
	Child mortality: relative ratio [wealthy quintile(I)/poor quintile(V)]		0.5	0.2	0.3	0.8	(31)	1996–2006
	Percent population with 1 dose measles vaccination: absolute difference by wealth quintile [wealthy quintile (I)/less poor quintile (V)]		24.7	13.6	1.9	46.9	(31)	1996–2006
	Percent population with 1 dose measles vaccination: relative ratio (wealthy quintile/poor quintile)		1.7	0.8	1	4.6	(31)	1996–2006
	Percent live births with skilled personnel: absolute difference by wealth quintile [wealthy quintile (I) less poor quintile (V)]		48.7	18.4	5.8	78.1	(31)	1996–2006
	Percent live births with skilled personnel: relative ratio [wealthy quintile (I)/poor quintile (V)]		6.3	8.2	1.1	38	(31)	1996–2006
Health and health care determinants – fixed context	Fixed context (*n*=57)							
	Accountability and voice (−2.5 to +2.5)		−0.07	0 .96	−2	1.6	(30)	2002
	Control in limited regressions: number of lower income countries (2002) (*n*)		23	n/a	n/a	n/a	(30)	2002
	Control in limited regressions: number of lower middle income countries (2002) (*n*)		13	n/a	n/a	n/a	(30)	2002
	Control in limited regressions: number of upper middle-income countries (2002) (*n*)		11	n/a	n/a	n/a	(30)	2002
	Control in limited regressions: number of high-income countries (2002) (*n*)		10	n/a	n/a	n/a	(30)	2002
Health and health care determinants – intermediate	Intermediate factors (*n*=57, except poverty)							
	Access to improved drinking water (%)		92	12	40	100	(31)	2000
	Education (mean number of years)		7.1	3	1	12.4	(30)	2000
	Percentage of the population below the national poverty line (%) (*n*=34) (2000–2006)		37	15	6	69	(30)	2000–2006
	Determinants equity measures (*n*=23)							
	Absolute difference in access to improved sources of drinking water (urban–rural) (*n*=23)		27	17	−6	70	(31)	2000
	Gini coefficient [0–1 index (1 – highest income inequality)]		0.43	0.9	0.3	0.64	(30)	2000–2005
Responsiveness	Aggregate level – percentage of responsiveness problems (%)	Dignity (*n*=57) Prompt attention (*n*=57)	22 35	11 12	6 16	53 67	(35 35)	2002/2003 2002/2003
	Inequality in responsiveness: difference by wealth or levels of responsiveness problems in poorest quintiles (IV, V) (outpatient services) (%)	Responsiveness level of problems in the poor quintile (I, II) (*n*=25)	40	9	28	59	(35)	2002/2003
		Absolute difference [wealthy (V)/less poor (I, II)] (*n*=25)	0	6	0	22	(35)	2002/2003
		Relative ratio (wealthy/poor) (*n*=25)	2	0	0	2	(35)	2002/2003
Healthcare resources and financial protection (for medical costs)	Health expenditure per capita (International Dollars) (*n*=57)		624	837	21	3,409	(34)	2002
	Out-of-pocket health expenditure as a percentage of total health expenditure per cap (*n*=25)		47	18	3	71	(30)	2002
Population demographics and prevalent diseases	Population more than 60 years (%) (2006) (*N*=57)		11	7	2	24	(31)	2006

a All wealth inequalities are based on household asset index quintiles (country-specific) calculated and provided by the data source listed.

Acquiring and linking data from different sources took place between August and December 2014. Two consolidated data sets were used for analyses. The final six health and coverage average levels data set contained between 52 (coverage) and 57 (health) country-level records. The final three data sets for health and coverage inequalities consisted of 23 (country-level) records each.

Responsiveness indicators were derived from health service user responses to the WHS for all 57 countries as indicated earlier. Responsiveness level indicators were calculated by averaging domain summations of individual-level responses dichotomized from a five-point verbal response scale (‘very good’, ‘good’ [0, no problem] ‘moderate’, ‘bad’, and ‘very bad’ [1, problem]). Dichotomizing the scale and standardizing by education and self-reported health status make results less susceptible to ‘reporting behaviour’ bias and more comparable across countries (36). The final indicator calculated for the average level of responsiveness was: the frequency of reporting ‘a problem’ or ‘poor responsiveness’ in a particular domain. The domains of prompt attention and dignity were selected as they were among the two most important domains across a wide range of countries (37), and illustrated two different faces of responsiveness as described in the original WHO work (1): prompt attention, ‘client orientation’ domain, and dignity, a ‘respect for persons’ domain. A composite responsiveness equity indicator was used for outpatient services rather than having domain-specific indicators. The responsiveness equity indicator was the average percentage across domains of responsiveness problems reported in the bottom two wealth quintiles for the less healthy in the population (those reporting moderate, poor, or very poor health). Because of small numbers, the bottom two wealth quintiles were used rather than just the bottom. The wealth quintiles were based on cross-country comparable asset indices and made available by WHO as part of the World Health Survey data set (38). Like the poverty measure, this is not strictly an inequality measure. However, it does measure the responsiveness experiences of disadvantaged groups, which could explain inequities in health and coverage outcomes. Neither the relative or absolute gap measures of inequality for responsiveness showed any correlation with the dependent variables.

#### Missing data procedures

Missing data were not extensive for the final analyses. The missing data procedure followed used multiple imputation by chained equations as specified in the standard Stata mi command routines and associated instructions (39). Missing data for the dependent (health and coverage) country-level indicators were not filled and the procedure was not necessary for the responsiveness and health systems indicators. Missing data for the determinants indicators were predicted from the country income group (dummy) and total health expenditure per capita. The following variables and observations were incomplete before imputation: accountability and voice index (missing for Cote d'Ivoire, Congo, Sri Lanka); and mean years of schooling for the population of 15 years or more in 2000 (which of the variables filled had the highest missing rates, for 9 out of 57 countries: United Arab Emirates, Bosnia and Herzegovina, Congo, Georgia, Israel, Lao People's Democratic Republic, Myanmar, Senegal, and Tunisia). The percentage of the population below the national poverty line in 2002 was only available for 35 countries out of 57 countries and therefore not filled.

### Analyses

Standard univariate and bivariate descriptive analyses on the dependent and independent variables preceded regression analysis (see Appendix 2). Normality of the distribution was tested. With respect to dependent variables, distributional characteristics required several transformations. The logarithmic transformation of the dependent variables generally improved analytical properties. It was necessary to log the mortality rates in order to normalize the skewed data distribution (16, 40, 41). For predictor variables, health expenditure per capita and the difference in access to improved water sources also required log transformation. These transformations do not affect the principle relationships tested. Scatter plots were used to assess the linearity of bivariate associations between predictor and outcome variables. Correlation matrices were used to assess collinearity of predictors (the highest correlation was a Pearson correlation coefficient of 0.75 for access to improved water and log health expenditure per capita).

Different regression models were tested: ordinary least-squares (OLS) linear regression, OLS log-linear regression, and Poisson and negative binomial maximum likelihood regressions. Although OLS regressions are more common than Poisson-based models, it was appropriate to try different models based on assumptions regarding the outcome variable (42). Judging the appropriate form of the model of the outcome variables required assessing model fit statistics, considering the underlying data generation mechanisms assumption, as well as *a priori* assumptions regarding the impact of predictors on outcomes variables. Model comparisons were undertaken for the domains of dignity, prompt attention, and basic amenities as these variables have high importance and variance across countries (24, 37). Model comparisons for mortality outcome variables, including log-linear regressions and Poisson-based negative binomial models. The negative binomial is a form of the Poisson that recognizes the original count, and integer (non-negative) nature of data, while relaxing assumptions regarding the mean equal to the variance (high mean dispersion). It is arguably preferred for mortality regressions (42, 43). Whereas OLS model fit statistic uses *R*^2^, which ranges from 0 to 1, with numbers closer to 1 representing higher fit, the log-likelihood becomes more positive as fit improves. Comparing models using the log-likelihood statistic requires calculation of the likelihood ratio chi-squared test (−2 times the difference in the log-likelihood ratios between the baseline and fitted models).

Only negative binomial regressions were used in regressions on average levels of health – maternal mortality, child mortality, and Tuberculosis (TB) cause of death (mortality). For the aggregate levels of health coverage–population coverage of births by skilled attendants, coverage with measles vaccination, and receiving Pap smears – linear log and linear regressions were used. Final regression models for health outcomes and coverage levels contained a total of six predictor variables (after poverty rate was tested initially) and were each run twice in order to have separate predictions for dignity and prompt attention. This was done to reduce variables in a single regression, given the sample sizes of 57 and high correlations between responsiveness domain scores (Pearson correlation coefficient, 0.85).

For regressions on inequalities, final models had only four predictors at a time (only 23 countries). To select the four predictors, once again, the pretesting of several models was performed. Regression results shown are selected from the model with the highest *R*^2^ or the most positive log-likelihood ratios (best fit) from the three combinations of independent variables tested, which were: 1) out-of-pocket expenditure, responsiveness inequality, difference in access to drinking water between urban and rural areas, accountability, and voice, Gini (largest number of variables); 2) out-of-pocket expenditure, responsiveness inequity, years of education (smallest number of variables); and 3) responsiveness inequity, the difference in access to drinking water between urban and rural areas, years of education, Gini. In all regressions, a larger number meant greater inequity (favouring wealthier). In results, regressions were presented for difference and ratio properties of the three dependent variables (six regression results).

Coefficients were assessed for statistical significance at the intervals: <0.10; <0.05; and <0.001.

The negative binomial regression coefficients were interpreted as an increase of *x* in an explanatory variable multiplying the fitted mean mortality rate by exp(*bx*) (42).

## Results

### Comparing regression models for the role of predictors of health outcomes

Table 2 displays the regression test results using maternal mortality with the dignity domain for responsiveness as an example. Four regression formats are shown: OLSs linear and log-linear models (models 1–3), Poisson and negative binomial models (models 4–6). Comparisons of this nature were made for all outcome variables.

**Table 2:** Maternal mortality cross-country regression models using the responsiveness dignity domain only (percentage of problems reported by health service users)^a^

Regression no.	Model 1	Model 2	Model 3	Model 4	Model 5	Model 6	
	
Model	Ordinary least-squares regression	Log-linear ordinary least-squares regression	Log-linear ordinary least-squares regression (with poverty)	Basic Poisson regression	Negative binomial maximum likelihood regression	Negative binomial maximum likelihood regression	Comparing coefficient in models 2 and 6
Fit							
MSE	190.8	0.82	0.93				
*R*^2^ (or pseudo)	0.76	0.86	0.5	0.93	0.17	0.14	
Log-likelihood				−6,776	−359	−373	
Average total health expenditure per capita (log)							
Coefficient	−19.54	−0.4	−0.03	0.18	0.02	−0.26	−0.40; −0.26
Std error	38.36	0.16	0.14	0.01	0.09	0.12	
*T*-statistics	−0.51	−2.42	−0.21	35.14	0.22	−2.17	
*p*	0.61	0.02	0.84	0	0.83	0.03	
Percent population with responsiveness problems							
Coefficient	110.39	0.33	0.34	−0.17	0.31	0.29	0.33; 0.29
Std error	46.84	0.2	0.16	0.01	0.11	0.14	
*T*-statistics	2.36	1.63	2.09	−32.28	2.75	1.97	
*p*	0.02	0.11	0.05	0	0.01	0.05	
Percent population accessing drinking water							
Coefficient	−592.68	−0.81	−0.24	−0.42	−0.21	−0.48	−0.81; −0.48
Std error	133.13	0.57	0.44	0.01	0.32	0.46	
*T*-statistics	−4.45	−1.42	−0.55	−42.29	−0.66	−1.04	
*p*	0	0.16	0.59	0	0.51	0.3	
Accountability and voice							
Coefficient	74.11	0.01	−0.09	0.25	−0.06	−0.04	0.01; −0.04
Std error	41.4	0.18	0.18	0	0.12	0.13	
*T*-statistics	1.79	0.04	−0.51	92.95	−0.52	−0.3	
*p*	0.08	0.97	0.61	0	0.6	0.77	
Average years of schooling							
Coefficient	−28.53	−0.05	−0.1	−0.05	−0.11	−0.07	−0.05; −0.07
Std error	12.85	0.06	0.05	0	0.03	0.04	
*T*-statistics	−2.22	−0.86	−1.99	−34.89	−3.28	−1.73	
*p*	0.03	0.4	0.06	0	0	0.08	
Percentage population over 60 years							
Coefficient	−13.51	−0.15	−0.14	−0.25	−0.17	−0.18	−0.15 −0.18
Std error	6	0.03	0.03	0	0.02	0.02	
*T*-statistics	−2.25	−5.84	−4.65	−151.75	−10.01	−8.67	
*p*	0.03	0	0	0	0	0	
Percent population below national poverty line coefficient							
Coefficient			0.03				
Std error			0.01				
*T*-statistics			3.4				
*p*			0				
Income group (low-middle)							
Coefficient				−0.91	−0.95		
Std error				0.01	0.25		
*T*-statistics				−89.84	−3.74		
*p*				0	0		
Income group (middle)							
Coefficient				−1.73	−1.97		
Std error				0.02	0.34		
*T*-statistics				−107.24	−5.83		
*p*				0	0		
Income group (high)							
Coefficient				−2.71	−2.77		
Std error				0.09	0.46		
*T*-statistics				−30.57	−6.05		
*p*				0	0		
Constant							
Coefficient	3530.55	12.66	7.61	−2.4	−1.27	−0.48	
Std error	515.92	2.21	1.91	0.03	1.23	1.78	
*T*-statistics	6.84	5.72	3.98	−75.58	−1.03	−0.27	
*p*	0	0	0	0	0.31	0.79	

a *N* is 57 countries for all regressions except for Model 3, where the number of country observations is 34.

Comparing the model fit statistics for OLSs regression shows better fit for log linear regressions (model 2). The Poisson regression log-likelihood statistics indicate poor fit relative to the negative binomials (more negative). In the negative binomial regressions, compared with baseline models (containing only population over 60 years), both regression models likelihood ratio tests are adequate to warrant inclusion of more predictors (*p*<0.000).

Specific experimentation showed that for all models, the percentage of the population older than 60 years is significantly associated with the level of maternal mortality. This obvious demographic–biological need pathway will receive no further comment. Other variables show less uniform patterns.

Using OLS regression (regression 1), lower maternal mortality, without log transformation, is predicted by responsiveness but not by health expenditure per capita. This result is contrary to theory-driven expectations. In regression 2, with outcome variables log transformed, there is an association of maternal mortality with total health expenditure per capita (natural log), but the association for responsiveness is small and non-existent for years of schooling and access to drinking water. Model 3, which also treats the outcome variable as logged, adds as an independent variable, the percentage of the population below the national poverty line, which was available for 34 mostly lower and lower middle income countries out of 57. National poverty rates are associated with maternal mortality [coefficient 0.03 (*p*=0.00)] as would be expected. Again, the effect of national poverty rates swamps out health expenditure per capita and accountability, which may suggest that the log-transformation alone is insufficient to correct for the underlying distributional form.

Regression 5 introduces the country's average level of income (World Bank categories) as independent variables. Income removes some effects of other variables, in particular for health expenditure per capita, responsiveness, and years of schooling. Regression 6, on the other hand, shows up these variables. Maternal mortality across countries is associated with health expenditure (coefficient: 0.26; *p*=0.03), with responsiveness barriers (coefficient: 0.29; *p*=0.05), and average years of schooling (coefficients: −0.07; *p*=0.08). Although associations with accountability and access to water and sanitation are insignificant, the clearer pathways related to health systems and health service interactions make this model preferable in our view, given that the health outcome maternal mortality is usually associated with the lack of health service attention at birth.

### Regression models that explain aggregate health levels

Six regressions of health outcomes and coverage rates are shown in Tables 3 and 4 for two domains: 1) dignity, and 2) prompt attention. Regressions otherwise contain the same independent variables. The first regression column shows the maternal mortality negative binomial regression with dignity (repeated from Table 2, Model 6). The next column in Table 3 shows the regression for child mortality, then TB mortality rates, and so on.

**Table 3:** Cross-country regression models for health outcomes and health service coverage, using the responsiveness domain dignity only^a^

	Health outcomes			Service coverage		
	
Explanatory variables	Maternal mortality (natural log by negative binomial model)	Child mortality (natural log by negative binomial model)	TB mortality (natural log by negative binomial model)	Percentage coverage by skilled attendants at birth (natural log)	Percentage coverage of measles vaccination (natural log)	Percentage coverage of Pap smear
Health expenditure per capita (log)	−0.26**	−0.21**	−0.2	0.48**	−0.15	9.24**
Users reporting responsiveness problems	0.29**	0.21*	0.74***	−0.49**	−0.25	−6.79
Access to improved drinking sources/water	−0.48	−0.75*	−0.69	2.53***	1.22	−8.06
Accountability and voice (−2.5 to +2.5) higher better	−0.04	−0.18*	−0.19	−0.32	0.05	−0.97
Average years of schooling of adults (>18 years)	−0.07*	−0.04	0.07	0.11	0.16**	1.14
Percentage of population over 60 years of age	−0.18***	−0.07***	−0.11***	0.1***	0.05	1.12*
Constant	−0.48	2.56*	−2.87	−14.03***	−4.36	−14.83
Model fit	Negative binomial	Negative binomial	Negative binomial	Log-linear	Log-linear	Ordinary least squares
MSE				1.01	1.13	18.53
*R*^2^ (pseudo for Poisson, NB)	0.16	0.09	0.07	0.81	0.37	0.59
Log likelihood	−359	−563	−503			

a *n*=57 except for regression for skilled birth attendants (*n*=52).

* *p*<0.10

** *p*<0.05

*** *p*<0.001.

**Table 4:** Cross-country regression models for health outcomes and health service coverage, using the responsiveness domain prompt attention only^a^

	Health outcomes			Service coverage		
	
Explanatory variables	Maternal mortality (natural log by negative binomial model)	Child mortality (natural log by negative binomial model)	TB mortality (natural log by negative binomial model)	Percentage coverage by skilled attendants at birth (natural log)	Percentage coverage of measles vaccination (natural log)	Percentage coverage of Pap smear
Health expenditure per capita (log)	−0.28**	−0.22**	−0.22	0.55***	−0.04	10.61***
Users reporting responsiveness problems	0.31*	0.22	0.83***	−0.34	0.23	−2.52
Access to improved drinking sources/water	−0.5	−0.76**	−0.53	2.6***	1.22	−7.28
Accountability and voice (−2.5 to +2.5) higher better	−0.08	−0.21**	−0.26	−0.25	0.05	−0.26
Average years of schooling of adults (>18 years)	−0.07*	−0.04	0.06	0.1	0.15**	1.03
Percentage of population over 60 years of age	−0.18***	−0.07***	−0.11***	0.1***	0.05	1.1*
Constant	−0.52	2.5*	−3.71	−14.14***	−4.48	−16.81
Model fit	Negative binomial	Negative binomial	Negative binomial	Log-linear	Log-linear	Ordinary least squares
MSE				1.03	1.13	18.89
*R*^2^ (pseudo for Poisson, NB)	0.16	0.09	0.07	0.8	0.37	0.57
− 2 times the log likelihood	−359	−563	−503			

a *n*=57 except for regression for skilled birth attendants (*n*=52).

* *p*<0.10

** *p*<0.05

*** *p*<0.001.

Across Tables 3 and 4, one observes that statistically significant coefficients for predictors are more common for mortality rate regressions than for coverage rate regression. In Tables 3 and 4, column 3, there are a high number of significant covariates, in the expected direction, for child mortality on the one hand (all predictors except adult education) and a low number for measles coverage on the other hand (Tables 3 and 4, column 6). Responsiveness is statistically significant for all mortality regressions but for only one of the service coverage (skilled attendants). Higher percentages of responsiveness problems in countries are associated with increased maternal, TB, and injuries mortality (Table 3, columns 2–4 and Table 4, columns 2 and 4), and reduced coverage of the population with skilled birth attendants (Table 3, column 5). On average, the effect sizes of responsiveness on the dependent variable, measured in terms of numerical percentages, are higher for service coverage than for mortality rates. Using coefficient results in Table 2, and the proportionate formula for interpreting negative binomial regression coefficients, as described in the methodology [exp b(x)=exp(0.29*0.10)], an increase in responsiveness problems by 10% increases mortality rates by 3% and decreases service coverage rates by 5%.

Looking across indicators, access to improved drinking sources is statistically significant in only one mortality regression – child mortality – and one coverage regression – skilled attendants at birth. Accountability (and voice) is statistically significant only in one mortality regression – child mortality. Average years of schooling is relevant to one mortality outcome – maternal mortality – and to one coverage outcome – measles vaccination. Health expenditure per capita is statistically significant in four out of six regressions (except TB mortality and measles coverage).

Model fit within health outcomes regressions, as judged by log-likelihood statistics, is best for maternal mortality (LL=−359), followed by TB mortality (LL=−503) and child mortality. In health service coverage regressions, fit as judged by the *R*^2^ statistic is better for skilled attendants at birth (*R*^2^=0.81) and Pap smear (*R*^2^=0.55) than measles vaccination (*R*^2^=0.37).

Qualitative changes are observed in the effects of dignity versus prompt attention. For child mortality, prompt attention barriers are not significant, whereas dignity barriers are significant (*p*<0.05). On the other hand, both dignity and prompt attention barriers are highly significant (*p*<0.001) for TB mortality rate regressions. However, effect sizes for TB are larger for prompt attention responsiveness barriers than for dignity.

### Regression models that explain aggregate health inequalities

Table 5 shows regression results for health outcome inequalities and service coverage inequalities as dependent variables. Child mortality favoured combinations of variable sets 1 and 3, whereas coverage inequality models were best fitted with the smaller set of predictor variables from set 2.

**Table 5:** Cross-country regressions explaining inequalities in health status and health service coverage by contextual and instrumental factors including responsiveness (*n*=23)

	Child mortality rates		Births attended by skilled personnel		Measles vaccination coverage	
	
	Difference ABS (poor-rich), larger worse	Ratio (poor/rich), larger worse	Difference (rich – poor), larger worse	Ratio (rich/poor), larger better	Difference (rich - poor), larger worse	Ratio (rich/poor), larger worse
Model	3	1	1	2	2	2
Out-of-pocket health expenditure		0.01	0.04	0.01	0.27**	0.01**
Responsiveness problems (% unhealthy, poor)	0.02	−0.49	0.81	0.11	0.31	0.01**
Difference in percent population accessing drinking water	0.01	0	−0.1			
Accountability index		0.50 (0.15)	14.98 (0.12)			
Average years of schooling	0.11			−1.54**	−2.72**	−0.07**
GINI (0–1, 1 unequal)	0.05**	0.02	−0.9			
Model fit	Negative binomial	Ordinary least squares	Ordinary least squares	Ordinary least squares	Ordinary least squares	Log linear
MSE		0.74	20.43	7.46	9.53	0.24
*R*^2^ (pseudo)	0.05	0.11	0.03	0.15	0.5	0.55
Log likelihood	−118.09					

* *p*<0.10

** *p*<0.05

*** *p*<0.001.

**Appendix 1:** Countries in regression analyses: outcome variables for health status and coverage

Country	Regression 1: maternal mortality per 100,000 lbs	Regression 2: child mortality per 1,000 live births*	Regression 3: TB cause of death per 100,000	Regression 4: percentage of births attended by skilled health personnel – 2000–2006 (2008 WHS)*	Regression 5: percentage of population with coverage with one dose of measles vaccination in the first year of life – 2003 (WHS 2005)*	Regression 6: percentage of women receiving a Pap smear (2000–2006) (WHS 2008, 58 countries)
Bangladesh	570	69	4	20	77	0
Bosnia and Herzegovina	3	17	6	100	84	40
Brazil	110	35	7	97	99	72
Burkina Faso	700	207	54	54	76	5
Chad	1,500	200	82	14	61	6
China	45	37	16	98	84	21
Comoros	400	73	7	62	63	8
Congo	740	108	70	83	50	23
Cote d'Ivoire	810	193	104	57	56	7
Croatia	7	7	6	100	95	65
Czech Republic	4	5	1	100	99	73
Democratic Republic	660	91	25	19	42	3
Dominican Republic	150	35	16	96	79	66
Ecuador	210	27	25	80	99	45
Estonia	25	8	6	100	95	53
Ethiopia	720	169	79	6	52	1
Finland	7	4	1	100	97	67
France	8	5	1	Not available	86	75
Georgia	66	45	13	92	73	13
Ghana	560	95	50	50	80	3
Hungary	6	9	3	100	99	65
India	450	87	30	47	67	3
Ireland	1	6	1	100	78	39
Israel	4	6	1	Not available	95	45
Kazakhstan	140	73	20	100	99	79
Kenya	560	123	133	42	72	4
Latvia	10	13	10	100	99	3
Luxembourg	12	4	1	100	91	82
Malawi	1,100	178	97	54	77	3
Malaysia	62	7	16	100	92	30
Mali	970	220	73	41	68	5
Mauritania	820	184	60	53	71	4
Mauritius	15	17	11	99	94	13
Mexico	60	28	4	94	96	64
Myanmar	380	106	20	57	75	1
Namibia	210	65	81	76	70	13
Nepal	830	82	23	19	75	3
Norway	7	4	1	Not available	84	73
Pakistan	320	103	40	54	61	3
Paraguay	150	29	12	100	91	53
Philippines	230	36	48	60	80	10
Portugal	11	6	4	100	96	59
Russian Federation	28	16	21	100	96	78
Senegal	980	137	52	52	96	11
Slovakia	6	8	3	100	99	59
South Africa	400	66	134	92	83	6
Spain	4	5	2	Not available	97	60
Sri Lanka	58	15	9	97	99	2
Swaziland	390	153	269	74	94	62
Sweden	3	4	0.5	Not available	94	70
Tunisia	100	24	2	90	90	10
Ukraine	18	20	16	100	99	34
United Arab Emirates	37	8	2	100	94	12
Uruguay	20	15	3	100	95	62
Viet Nam	150	23	22	88	93	7
Zambia	830	182	138	43	84	3
Zimbabwe	880	126	131	69	80	9
Average	308	63	36	76	84	31
Std dev.	368	66	50	28	15	29
Minimum	1	4	1	6	42	0
Maximum	1,500	220	269	100	99	82

**Appendix 2:** Comparing univariate distributions for skewness and Kurtosis statistics: example for health outcomes

		Distribution	
		
Variables	Variable form logged	Skewness	Kurtosis
Maternal mortality rate per 100,000 live births (2005)	No	1.15	3.48
Natural log of maternal mortality rate	Yes	−0.35	1.78
Maternal mortality counts	No	4.79	25.24
Natural log of maternal mortality counts	Yes	−0.17	1.81
Under 5 child mortality per 1,000 live births (2005)	No	0.98	2.65
Natural log of child mortality rate	Yes	−0.12	1.64
Child mortality counts	No	4.77	27.11
Natural log of child mortality counts	Yes	−0.04	2.07
TB cause of death per 100,000 (2004)	No	2.32	9.66
Natural log of TB cause	Yes	−0.19	1.99

Responsiveness problems experienced by the unhealthy poor groups were statistically significant only for measles vaccination inequalities between rich and poor. Out-of-pocket expenditure was statistically significant in predicting coverage gaps for measles immunization. With respect to the broader determinants, the Gini coefficient mattered most for inequalities in child mortality between the rich and poor, and education mattered more for inequalities in births attended by skilled personnel.

## Discussion

This paper presents an exploration of different models for understanding the linkages between health and service coverage outcomes, and related health determinants, including health systems’ responsiveness. We used a set of cross-sectional analyses of different types of health status and health service coverage rates to explore different sets of determinants and health systems’ responsiveness indicators across 57 countries. To our knowledge, this is the first time that both health conditions and service coverage rates are explained using determinants and ‘acceptability’ barriers of responsiveness.

The determinants’ indicators tested here were associated with health in the expected directions as shown elsewhere (17, 20, 21, 40, 41), which is reassuring with respect to the findings for health systems’ responsiveness. An interesting new finding is that responsiveness was systematically associated with poorer health outcomes and coverage in the areas of maternal mortality, child mortality, TB mortality, skilled birth attendance coverage, and Pap smears (not measles vaccination). The results imply that both responsiveness barriers and health determinants have quantifiable, separate associations with health status and health service coverage. Responsiveness complements the financial barriers indicators recommended to be measured as part of UHC in the health goal, SDG-Goal 3.

Our analyses also have implications for monitoring health determinants in the SDGs. SDG-Goal 3 (health) covers health outcomes and ‘UHC’ (6, 12). UHC in the SDGs is defined as the degree to which health services meet population healthcare needs without undue financial hardship. Two metrics derived for its quantification are: financial protection coverage of individuals, which is measured by the absence of so-called catastrophic direct medical costs (5), and service or intervention coverage, which is measured as the proportion of people, who need particular well-accepted health interventions, receiving them. Both metrics can also be expressed as coverage inequality (by sex, education, income, and geographic area) (17). Yet, these metrics do not explicitly track responsiveness barriers, or the wider panorama of social and environmental determinants such as education of mothers and income inequality, which are clearly important for achieving good population health and effective health service coverage.

The systematic testing of regression models, variables, and indicators as illustrated in this paper, is useful for determining which national comparable health determinants indicators to track. Our findings show that several determinant indicators are candidates. These include drinking water coverage and coverage inequalities, poverty, mean years of schooling, and income inequality. These are candidates for both international and national use in intersectoral monitoring frameworks that track health determinants. Except for poverty, the data series are relatively complete (poverty is more complete now relative to 2002–2004) and they complement SDG-Goal 3 (health). These indicators are also likely to be used by sectors beyond health in monitoring other SDG goals (e.g. Goal 1 – poverty, Goal 4 – education, Goal 6 – water and sanitation, and Goal 10 – income inequality). Having the health sector in national contexts tracking a set of determinant indicators is vital, as described in the Health in All Policies approach (44). Tracking determinants is statistically simple as well as efficient and provides a rational for policy coherence if the same indicators are already being used by another sector to monitor their strategic performance. These data can also be used as a bridge to build better information systems for health impact assessments, thereby enabling anticipation of health changes before they emerge as behavioural changes in the population.

There are several limitations to our study. It consists of data that are 12–13 years old. It is possible that, as health systems and development contexts have changed, other patterns would have emerged if the study had been conducted on current data (e.g. governance accountability concepts can have altered). We also used a limited number of variables. More recommendations for the use of variables in future research are discussed below. A further limitation is that we only conducted relatively simple cross-sectional analyses, which yielded information on associations but specific longitudinal analyses should be investigated in the future for more causative tracking of health determinants. One example of a recent study that used more sophisticated mathematical underpinnings is Mondal and Shitan (45), which used path analysis and found a significant association for low and lower-middle income countries between life expectancy and mean years of schooling. In more complex methodological studies, there is a tendency for fewer health outcomes, predictors, and countries to be analysed due to data availability problems. Other typical enhancements to the analytical approach are time-series analyses (46) and multi-level analyses (20).

We were struck by the cross-country equity regression results. Although much is known about measuring and monitoring health and coverage inequalities (47), far less is known about the predictors of the aggregate levels of health inequalities. This is very important for understanding actions to improve health equity and which determinants to monitor. Currently, there is little empirical literature using country-level health inequalities metrics (48, 49) as dependent variables. Our paper used gap measures as dependent variables. Predictors were the Gini index, differences in drinking water access, and health systems responsiveness to poorer populations, which were all relevant, but not for all health and service conditions. For global monitoring of SDG-Goal 10, covering the reduction of inequalities within countries, it would be useful to know which determinants indicators are most closely linked to health inequalities.

In the future, a wider range of indicators could be tested for use in tracking health determinants as part of the SDGs. We selected what appeared to be feasible indicators, for some of which had available distributional information (i.e. for access to water). But several additional examples of theory-driven indicators were mentioned earlier. Variables already considered in the cross-country literature are female education (45). In our analysis, we used education overall, but further work would explore female education. Kolves et al. (50) used the Gini indices, unemployment rates, female participation in the labour force, GDP per capita, and divorce rates to predict suicide rates. Fritzell et al. (41) found child poverty rates and social spending were associated with child mortality. For our data set of 57 countries, poverty rates were too incomplete to use for all regressions. Another study showed that paid maternity leave was also associated with improved immunization coverage (48). These studies illustrate the more specific indicators that require further testing, including the importance of policy indicators. Data sets on policy indicators related to the labour market conditions for health may be of specific interest in this regard (see the World Policy Analysis Database: www.worldpolicycenter.org/).

A major obstacle to advancing empirical testing of determinants and barriers or facilitators of health services access, like responsiveness, is having both a holistic vision of health, and the available data (3). As part of the SDGs, the United Nations Secretary-General's Independent Expert Advisory Group on a Data Revolution for Sustainable Development encourages the collection of disaggregated data for monitoring equity across goals – most of which include important health determinants (51). The follow-up of these recommendations will be very important for any initiatives to track health determinants and their population health and health equity impacts. Investments need to be made to obtain better, disaggregated data about the real sector of the economy, societal well-being, and the environment (52). Health policymakers should advocate for better data collection and disaggregation in other sectoral indicators in order to identify common causes across sectors.

## Conclusions

In promoting monitoring of health determinants and related barriers to health service coverage like responsiveness, the health sector will enhance public health promotion, which is necessary for SDG-Goal 3, ‘attaining healthy life for all at all ages’ (5). It is only when national health monitoring by the health sector reflects the true intersectoral scope of health, that accountability across sectors for actions affecting health will be demanded by the whole-of-society.
